# Urocortin-1 promotes colorectal cancer cell migration and proliferation and inhibits apoptosis via inhibition of the p53 signaling pathway

**DOI:** 10.1007/s00432-024-05693-7

**Published:** 2024-03-28

**Authors:** Xiaolan Guo, Ya Li, Xiangyu Chen, Binghua Sun, Xiaolan Guo

**Affiliations:** Department of Gastroenterology, The First Affiliated Hospital of Zhengzhou University, Zhengzhou, Henan China

**Keywords:** Urocortin-1, p53, HCT-116, RKO, HT29, Colorectal cancer

## Abstract

**Purpose:**

To investigate the effect of urocortin-1 (UCN-1) on growth, migration, and apoptosis in colorectal cancer (CRC) in vivo and vitro and the mechanism by which UCN-1 modulates CRC cells in vitro.

**Methods:**

The correlation between UCN-1 and CRC was evaluated using The Cancer Genome Atlas (TCGA) database and a tissue microarray. The expression of UCN-1 in CRC cells was assessed using quantitative real-time polymerase chain reaction (RT-qPCR) and western blotting. In vitro, the influence of UCN-1 on the proliferation, apoptosis, and migration of HT-29, HCT-116, and RKO cells was explored using the celigo cell counting assay or cell counting kit-8 (CCK8), flow cytometry, and wound healing or Transwell assays, respectively. In vivo, the effect of UCN-1 on CRC growth and progression was evaluated in nude mice. The downstream pathway underlying UCN-1-mediated regulation of CRC was determined using the phospho-kinase profiler array in RKO cells. Lentiviruses were used to knockdown or upregulate UCN-1 expression in cells.

**Results:**

Both the TCGA and tissue microarray results showed that UCN-1 was strongly expressed in the tissues of patients with CRC. Furthermore, the tissue microarray results showed that the expression of UCN-1 was higher in male than in female patients, and high expression of UCN-1 was associated with higher risk of lymphatic metastasis and later pathological stage. UCN-1 knockdown caused a reduction in CRC cell proliferation, migration, and colony formation, as well as an increase in apoptosis. In xenograft experiments, tumors generated from RKO cells with UCN-1 knockdown exhibited reduced volumes and weights. A reduction in the expression of Ki-67 in xenograft tumors indicated that UCN-1 knockdown curbed tumor growth. The human phospho-kinase array showed that the p53 signaling pathway participated in UCN-1-mediated CRC development. The suppression in migration and proliferation caused by UCN-1 knockdown was reversed by inhibitors of p53 signal pathway, while the increase in cell apoptosis was suppressed. On the other hand, overexpression of UCN-1 promoted proliferation and migration and inhibited apoptosis in CRC cells. Overexpression of p53 reversed the effect of UCN-1 overexpression on CRC development.

**Conclusion:**

UCN-1 promotes migration and proliferation and inhibits apoptosis via inhibition of the p53 signaling pathway.

**Supplementary Information:**

The online version contains supplementary material available at 10.1007/s00432-024-05693-7.

## Introduction

The increasing incidence of colorectal cancer (CRC) makes it the third most common malignant tumor worldwide, which seriously endangers human health and increases the social burden (Hasbullah and Musa 2021). Early detection and treatment of CRC are important to effectively improve patient survival rate and quality of life (Lewandowska et al. 2022). CRC is usually diagnosed at an advanced stage (Or et al. 2016), because detection of the disease at early stage remains challenging. Systemic chemotherapy after surgical resection is generally administered to patients with advanced CRC. Clinical recurrence resulting from incomplete surgical resection and metastasis are the main causes of death related to CRC. Effective methods to inhibit CRC proliferation and migration to inhibit tumor recurrence after surgery and improve the patient survival rate are thus urgently needed (Peters et al. 2015).

Urocortin (UCN) belongs to the corticotropin-releasing-hormone (CRH) family and is involved in many physiological actions (Haznadar et al. 2018). It is widely expressed in the cardiovascular and central nervous systems, the pancreas, and digestive tracts of rats and humans (Wang et al. 2021; Zhou et al. 2022a, b; Gao et al. 2021; Vuppaladhadiam et al. 2020). UCN binds to G protein-coupled receptors, known as corticotropin-releasing factor receptors (CRF-Rs), to mediate stress responses (Haznadar et al. 2018), inflammation (Yang et al. 2008), and tumorigenesis (Cheng et al. 2014). Increasing evidence has shown the critical role of UCN in the development of diverse malignant tumors in humans. For example, UCN can affect the migration of hepatoma carcinoma (Wang et al. 2008; Zhu et al. 2014) and endometrial cancer (Florio et al. 2006). UCN has three isoforms, UCN-1–3. UCN-1, the first UCN discovered, plays a role in endometrial cancer progression (Owens et al. 2017), stress-induced gastrointestinal dysfunction (Ayyadurai et al. 2017), gastrointestinal motor function, and the processing of visceral pain (Martinez et al. 2004; Yin et al. 2008). A large body of literature supports the importance of UCN-1 in the intestinal tract (Tache et al. 2004; Koido et al. 2014), especially for stimulating colonic motor activity (Maillot et al. 2000, 2003; Martinez et al. 2002; You et al. 2015; Saruta et al. 2004). In contrast, the role of UCN-1 in CRC development has not been investigated.

In this study, the UCN-1 expression levels were evaluated using The Cancer Genome Atlas (TCGA) database and a tissue microarray. UCN-1 was strongly expressed in patients with CRC. The tissue microarray showed that high UCN-1 expression was associated with sex, higher risk of lymphatic metastasis, and later pathological stage. In CRC cells, we found that UCN-1 expression was upregulated. Lentivirus-mediated siRNA was used to knock down UCN-1, and the effects on CRC development in vitro and in vivo were analyzed. The results showed that downregulation of UCN-1 expression was critical to attenuate the proliferation and migration of CRC cells and increase apoptosis. We used the human phospho-kinase array to explore the downstream pathway of UCN-1 in regulating CRC and found that the p53 signal pathway participates in UCN-1-mediated CRC development. Inhibitors of the p53 signaling pathway reserved the effect of UCN-1 knockdown, while p53 overexpression reserved the effect of the upregulation of UCN-1 expression on CRC development. Therefore, UCN-1 may participate in the progression of CRC by inhibiting the p53 signaling pathway, which maybe a direction for the future treatment of CRC.

## Materials and method

### Cell culture

Fetal human colon cells (FHCs) and the human colon cancer cells HCT-116, RKO, and HT29 were purchased from the Cell Bank of the Chinese Academy of Sciences (Shanghai, China). The cells were cultured in RPMI 1640 with 10% fetal bovine serum (FBS) (both from Gibco, Grand Island, NY, USA), and 1% penicillin (100 U/ml)/streptomycin (100 µg/ml) at 37˚C in an atmosphere with 5% CO_2_.

### Human tissue microarray and immunohistochemistry

A total of 180 tissue samples (87 para-cancerous and 93 malignant CRC) were purchased from Shanghai Outdo Biotech (Shanghai, China). Tissue chip immunohistochemistry experiments were commissioned to BioSCI Res (Shanghai, China). All participants signed an informed consent form and provided the relevant clinical data. This study was conducted in accordance with the Code of Ethics of the World Medical Association (Declaration of Helsinki) for experiments involving humans. The experiments were approved by the Ethics Committee of the First Affiliated Hospital of Zhengzhou University (No. 2022-KY-0083-03).

The CRC tissue microarrays and xenograft tumor tissue slides were soaked in xylene and rehydrated in a series of alcohols. The tissue microarrays and slides were then repaired using citrate buffer, followed by blocking with H_2_O_2_ and serum. The specimens were incubated with primary antibodies against UCN-1 (1:100; 19731-1-AP; Proteintech) and Ki-67 (1:100; ab16667; Abcam) and secondary antibody (1:200; ab97080; Abcam). The tissue microarrays and slides were stained using DAB and hematoxylin and photographed. Two pathologists independently and randomly examined all specimens. The positive cell score was classified as 0 (0%), 1 (1–25%), 2 (26–50%), 3 (51–75%), or 4 (76–100%). The immunohistochemistry results were obtained by multiplying the positive cell score by staining intensity. A staining index < 6 was defined as low UCN-1 expression, while a staining index ≥ 6 as high UCN-1 expression.

### TCGA database analysis

Informative data on UCN-1 expression in CRC were obtained from TCGA (https://cancergenome.nih.gov/) and processed and analyzed using the R statistical software TCGAbiolinks.

### Cell transfection

To knock down UCN-1 in RKO cells, three short hairpin RNAs (shRNAs) were designed to target the UCN-1 gene: sh-RNA_1: 5′-GACAACCCTTCTCTGTCCATT-3′; sh-RNA_2: 5′-CGAGCAGAACCGCATCATATT-3′; and sh-RNA_3: 5′-CTCAATCTTGGACCGTACAGA-3′. They were inserted into the BR-V-121 lentiviral vector (Sangon Biotech, Shanghai, China). Non-silencing RNA (5′-TTCTCCGAACGTGTCACGT-3′) was used as negative control. The double-stranded shRNA was inserted into the plasmid containing the *GFP* gene and then linearized with EcoR I and Age I. Plasmids used to overexpress UCN-1 and p53 and the lentivirus packaging were also constructed by Sangon Biotech. Human embryonic kidney (HEK) 293T cells (American Type Culture Collection, Manassas, VA, USA) were co-transfected with different plasmids using Lipofectamine 2000 (Thermo Fisher Scientific, USA) following the instructions. After transfection, the cells were cultured for 48 h and then the lentivirus was harvested and concentrated. For lentivirus infection, HCT-116, RKO and HT29 cells were co-cultured with lentivirus-containing medium at a MOI of 10. Infection efficiency and knockdown or overexpression efficiency were evaluated using fluorescence microscopy, reverse transcription and quantitative real-time polymerase chain reaction (RT-PCR), and western blotting post-infection.

### RT-PCR assays

Total RNA was extracted from the cell lines using TRIzol (Sigma) according to the manufacturer’s instructions. The cDNA was synthesized from the total RNA using HiScript QRT SuperMix for qPCR (Vazyme). The relative gene expression level was measured using AceQ qPCR SYBR Green master mix (Vazyme) and quantified using the comparative 2 − ΔΔCt method. *GAPDH* mRNA was the endogenous control. The primer sequences were as follows: GAPDH forward primer 5′-TGACTTCAACAGCGACACCCA-3′, GAPDH reverse primer 5′-CACCCTGTTGCTGTAG CCAAA-3′; UCN-1 forward primer 5′-CGGGACAACCCTTCTCTGTC-3′; and UCN reverse primer 5′-TGCCCACCGAGTCGAATATG-3′.

### Western blotting

Protein lysates were obtained from the cells using RIPA lysis buffer with protease inhibitors on ice. The BCA Protein Assay Kit (HyClone-Pierce) was used to determine the total protein concentration. Equal quantities of denatured protein were subjected to SDS-PAGE and transferred to PVDF membranes. After blocking with 5% skim milk for 1 h, the PVDF membranes were incubated with specific primary antibodies: anti-UCN-1 (1:500; 19731-1-AP; Proteintech), anti-GAPDH (1:30000; 60004-1-lg; Proteintech), anti-p53 (1:2000; 10442-1-AP; Proteintech), and anti-p-p53 (1:1000; 67900-1-Ig; Proteintech), anti-pERK1/2 (1:2000; 28733-1-AP; Proteintech), anti-p-STAT3 (1:3000; ab76315; abcam), anti-STAT3 (1:3000; 60199–1-lg; Proteintech), anti-p-STAT1 (1:2000; ab109421; abcam), anti-STAT1 (1:1000; ml155017; Proteintech). Subsequently, the membranes were washed and blotted with Goat Anti-Rabbit (1:3000; A0208; Beyotime) or Goat Anti-Mouse (1:3000; A0216; Beyotime) secondary antibodies. GAPDH was used as an internal control.

### Celigo cell counting assay

Lentivirus-infected HCT-116 and RKO cells were seeded in a 96-well plate with 3000 cells per well and cultured at 37°C with 5% CO_2_. The number of cells was continuously evaluated using the Celigo imager (Nexcelom Bioscience, Lawrence, MA, USA) for green fluorescence for 5 days at the same time. The cell proliferation rate was analyzed based on the number of cells showing green fluorescence.

### Cell counting kit-8 (CCK‑8)

Lentivirus-infected HT-29, HCT-116, and RKO cells were seeded into 96-well plates at 5000 cells per well. After 24 h, the cells were treated with 20 µM Pifithrin-α (Selleck) or not, for another 24 h. Next, 10 μl of CCK‐8 (Sigma) solution was added into each well for 2 h at 37℃ before the assay. For the experiment of UCN-1 and p53 overexpression, 10 μl of CCK‐8 (Sigma) solution was added on 1th, 3th and 5th day. The optical density (OD) at 450 nm was measured to determine cell viability.

### Flow cytometry

Lentivirus-infected colon cancer cells were cultured in 6-well plates (2 mL/well). Then, cells from different treatments were collected and the cell precipitate was washed with D-Hanks (pH = 7.2–7.4) pre-cooled at 4°C. The Annexin V and PI Apoptosis Detection Kit (SouthernBiotech) was used to assess the cell apoptosis rates according to the kit protocol. Briefly, the collected cells were incubated with Annexin V-APC and PI for the indicated time at room temperature and detected using flow cytometry.

### Colony formation assay

Lentivirus-infected HCT-116 and RKO cells were cultured in 6-well plates (2 mL/well) at a density of 500 cells per well. The cells were cultured for up to 8 days and the medium was changed every 3 days. The visible colonies were fixed with 4% paraformaldehyde for 30–60 min and stained with 500 µl Giemsa for 10–20 min before being recorded using fluorescence microscope (Olympus, Tokyo, Japan).

### Wound healing assay

HCT-116 and RKO cells infected with lentivirus were seeded into 96-well plates at 50,000 cells per well with 100 μl of complete medium to each well. When the cell fusion reached approximately 90%, a scratch was made in the middle of the well. The cells were washed with PBS and incubated in a serum-free medium. The cells were cultured in an incubator at 37 °C with 5% CO_2_. Wound healing was observed under a microscope at 0 and 24 h. Wound closure was calculated using ImageJ 7.0.

### Cell migration assay

HT-29, HCT-116, and RKO cells from different groups in the logarithmic growth phase were seeded in a Transwell upper chamber (Corning Incorporated, Corning, NY, USA) inserted in 24-well plates, at a density of 50,000 per well. The Transwell chambers were coated with an 8.0-μm polycarbonate membrane. A total of 100 μL cell suspension with FBS-free medium was supplemented into the apical chamber, and 600 μl of RPMI 1640 medium containing 30% FBS was added to the basolateral chamber. After incubation for 48 h, the cells adhering to the lower surface were fixed with 4% paraformaldehyde, stained with Giemsa, and photographed under a microscope.

### Xenograft animal model

Nude mice (8 weeks, male) were obtained from GemPharmatech (Nanjing, China). The authors were accountable for all aspects of the work, ensuring that questions related to the accuracy or integrity of any part of the work were appropriately investigated and resolved. The experiments were performed under a project license (No. 2022-KY-0083-03) granted by the Animal Ethics Committee of The First Affiliated Hospital of Zhengzhou University. The animals were maintained in a sterile environment, and their cages, food, and bedding were sterilized using autoclaving. The mice had free access to food and fresh water. RKO cells infected with shCtrl or shUCN-1 were subcutaneously implanted into the right forelimb armpit of the nude mice. Each group included 10 mice. Tumor weight and growth in the post-injected mice were measured at indicated times. The tumor volume was calculated using the following formula: π/6 × L × S × S, where L represents the long axis and S, the short axis. Twenty-one days after injection, all mice were euthanized and tumor samples were collected for further analysis.

### Human Phospho-Kinase Array (proteome profiler)

The Human Phosphorylation Array was used to explore the effects of the downregulation of UCN-1 expression on phosphorylation-related proteins in RKO cells with and without UCN-1 knockdown. The Human Phospho-Kinase Array Kit (R&D Systems, Minnesota, USA) was used following the manufacturer’s protocol. The cell lysates were incubated overnight with the array membranes. The arrays were then washed and incubated with the detection antibody cocktails. Finally, the membranes were incubated with streptavidin conjugated to horseradish peroxidase, followed by chemiluminescence detection.

### Statistical analysis

All data were analyzed using GraphPad Prism 8. Expression patterns in CRC and para-carcinoma tissues were analyzed using the sign test. The relationship between UCN-1 expression and tumor characteristics in patients with CRC was analyzed using the Mann–Whitney U and Spearman rank correlation analyses. The data were analyzed using the t test, one-way analysis of variance (ANOVA) with Tukey’s test, and two-way ANOVA with Tukey’s post hoc multiple comparisons. The data are presented as the mean ± SEM from at least three independent replications. *P < 0.05, **P < 0.01, and ***P < 0.001 were considered to indicate statistical significance.

## Results

### The expression of UCN-1 was upregulated in CRC tissues

**Fig. 1:**
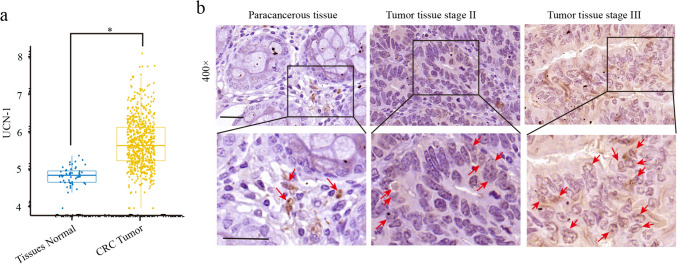
UCN-1 expression in CRC tissues. **a** The expression of UCN-1 in colorectal cancer tissue and para-cancerous tissue based on TCGA database. **b** Representative IHC staining of UCN-1 in CRC para-cancerous tissues and tumor tissues. Positive cells were indicated by red arrows. Scare bar, 100 μm

To assess the clinical relevance of UCN-1 in CRC, we first analyzed the TCGA data (number of CRC tissues: 635; para-cancerous normal tissues: 51). The bioinformatics analysis showed that the mRNA expression of UCN-1 significantly increased in CRC tissues (Fig. 1a) compared with that in normal tissues. A microarray that included 93 CRC tissues and 87 para-cancerous normal tissues was analyzed next. Compared with the para-cancerous normal tissues, most tumor tissues were immunoreactive for UCN-1 (Fig. 1b). Overall, 51 of 93 (54.8%) cases presented high UCN-1 expression in CRC tissues, whereas 3 of 87 (3.4%) cases showed high UCN-1 levels in para-carcinoma tissues (Table 1). According to the sign test, the expression of UCN-1 in the CRC tissues was significantly higher than that in the para-cancerous tissues (Table 1), indicating that UCN-1 can be used for subsequent statistical analysis of clinicopathological data such as tumor diameter, infiltration, lymph node metastasis, and clinical stage. According to the Mann–Whitney U analysis of the tissue microarray data, there was a significant difference in sex, tumor lymph node metastasis, and clinical stage between the groups with low and high UCN-1 expression (Table 2). Based on this result, we performed a Spearman rank correlation analysis for lymph node metastasis, sex, and clinical stage. We found that patients with CRC with abundant expression of UCN-1 were characterized by higher risk of lymphatic metastasis and later pathological stage than those patients with low UCN-1 levels (Table 3). Interestingly, high expression of UCN-1 was more likely in male patients (Tables 2 and 3). These results imply that UCN-1 may act as a cancer-promoting factor in CRC.

**Table 1:** Expression patterns in colorectal cancer tissues and para-carcinoma tissues revealed in immunohistochemistry analysis

UCN-1 expression	Tumor tissue		Para-carcinoma tissue		*p* value
	Cases	Percentage (%)	Cases	Percentage (%)	
Low	42	45.2	84	96.6	< 0.0001***
High	51	54.8	3	3.4	

***p < 0.001

**Table 2:** Relationship between UCN-1 expression and tumor characteristics in patients with colorectal cancer

Features	No. of patients	UCN-1 expression		*p* value
		Low	High	
All patients	93	42	51	
Age (years)				0.925
≥ 68	46	21	25	
< 68	47	21	26	
Sex				0.043*
Male	44	15	29	
Female	49	27	22	
Tumor size				0.828
≥ 5	37	17	20	
< 5	55	24	31	
Stage				0.001**
1	4	2	2	
2	46	28	18	
3	31	6	25	
Grade				0.706
1	1	0	1	
2	77	35	42	
3	14	6	8	
4	1	1	0	
T Infiltrate				0.356
T1	1	1	0	
T2	4	1	3	
T3	70	30	40	
T4	16	9	7	
Lymphatic metastasis (N)				< 0.0001***
N0	58	34	24	
N1	26	6	20	
N2	8	1	7	

*p < 0.05, **p < 0.01, ***p < 0.001

**Table 3:** Relationship between UCN-1 expression and tumor characteristics in patients with colorectal cancer

	UCN-1
lymphatic metastasis (N)	
Spearman coefficient	0.373**
Signification (double-tailed)	0.000***
*N*	92
Sex	
Spearman coefficient	– 0.211*
Signification (double-tailed)	0.043*
*N*	93
Stage	
Spearman coefficient	0.372**
Signification (double-tailed)	0.001**
*N*	81

*p < 0.05, **p < 0.01, ***p < 0.001

### Knockdown of UCN-1 decreased proliferation and migration but increased apoptosis of CRC cells in vitro

**Fig. 2:**
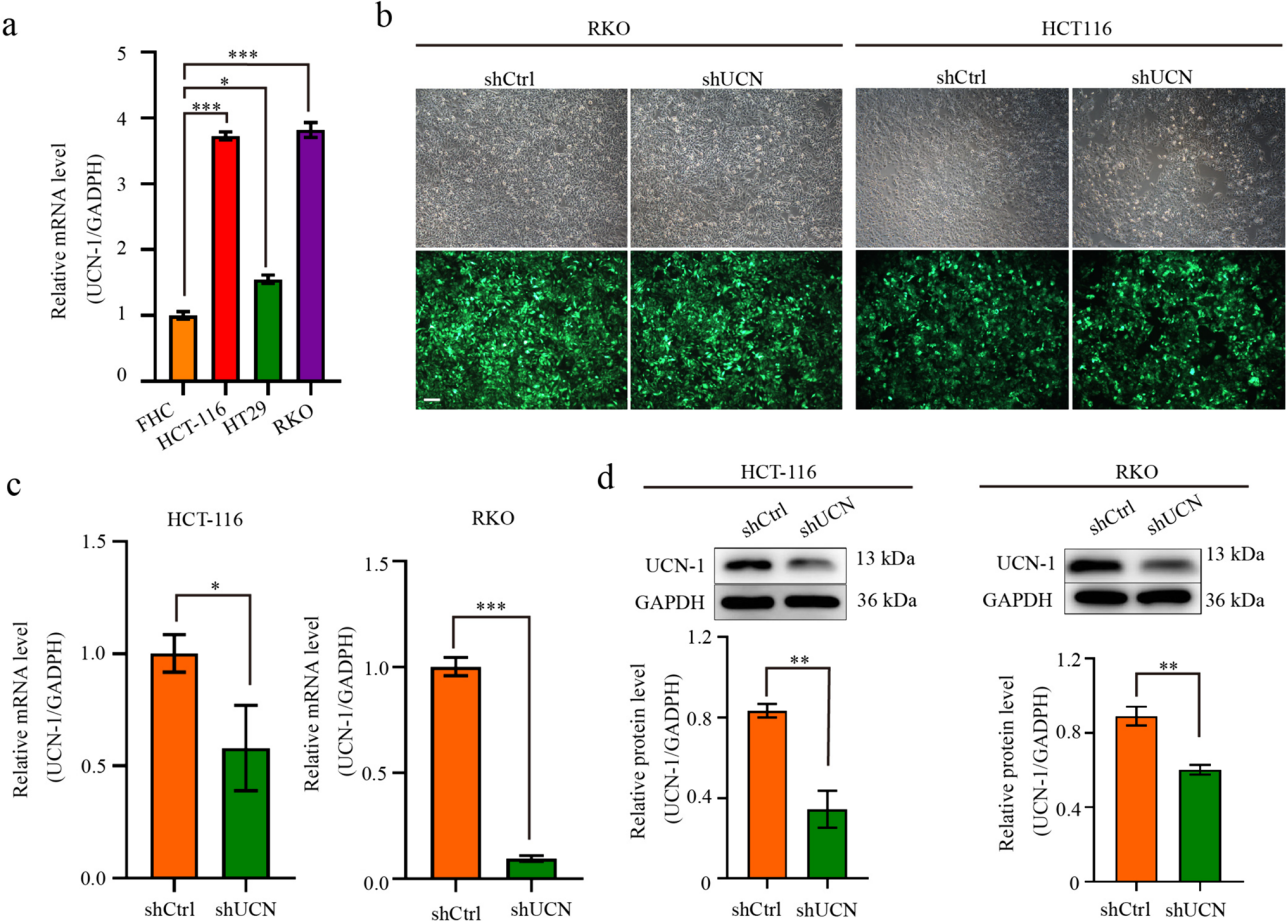
UCN-1 expression in CRC cell lines. **a** The mRNA expression of UCN-1 in FCH, HCT-116, HT29 and RKO cells. One-way ANOVA with multiple Tukey’s test. Data are presented as mean ± SEM. *P < 0.05, ***P < 0.001. *n* = 3. **b** Transfect efficiency of HCT-116 and RKO cells was evaluated by fluorescence imaging. Scare bar, 100 μm. **c** The mRNA expression of UCN-1 of HCT-116 and RKO cells after infection of lentivirus shUCN or shCtrl. *t* test. Data are presented as mean ± SEM. *P < 0.05. *n* = 3. **d** The protein expression of UCN-1 of HCT-116 and RKO cells after infection of lentivirus shUCN or shCtrl. t test. Data are presented as mean ± SEM. **P < 0.01. *n* = 3

Analysis of the clinicopathological data indicated that UCN-1 expression was related to CRC development. Next, we examined the expression of UCN-1 in CRC cell lines. We found that UCN-1 was highly expressed in different CRC cell lines—HCT-116, HT29, and RKO (Fig. 2a)—compared with its expression in the normal human colon cell line (FHC cells). Given that UCN-1 was abundantly expressed in the RKO and HCT-116 cell lines, we selected these two cell lines for subsequent experiments. As the expression of UCN-1 was positively correlated with tumor lymph node metastasis, UCN-1 may act as a promoting factor for CRC cell migration. To obtain evidence for this hypothesis, we downregulated UCN-1 expression using a lentiviral shRNA approach. Initially, RKO cells were transduced with three different shRNAs (shRNA_1, shRNA_2, and shRNA_3) that targeted UCN-1 (shUCN) or with a non-silencing control shRNA (shCtrl). Stable knockdown of *UCN-1* mRNA was achieved in the shRNA_2 group, whereas silencing was not successful in the shRNA_1 and shRNA_3 groups (Fig. S1). As shRNA_2 led to the best knockdown effect, we chose shRNA_2 to silence UCN-1 and study its function in the RKO and HCT-116 cell lines. To evaluate the infection efficiency of the shRNAs, a green fluorescent gene was linked to the shRNAs (Fig. 2b). Silencing UCN-1 did not affect RKO cell morphology. Furthermore, both the mRNA and protein expression of UCN-1 in the HCT-116 and RKO cells were detected. Our results showed that the expression of UCN-1 was significantly knocked down in the HCT-116 and RKO cells (Fig. 2c and d).

**Fig. 3:**
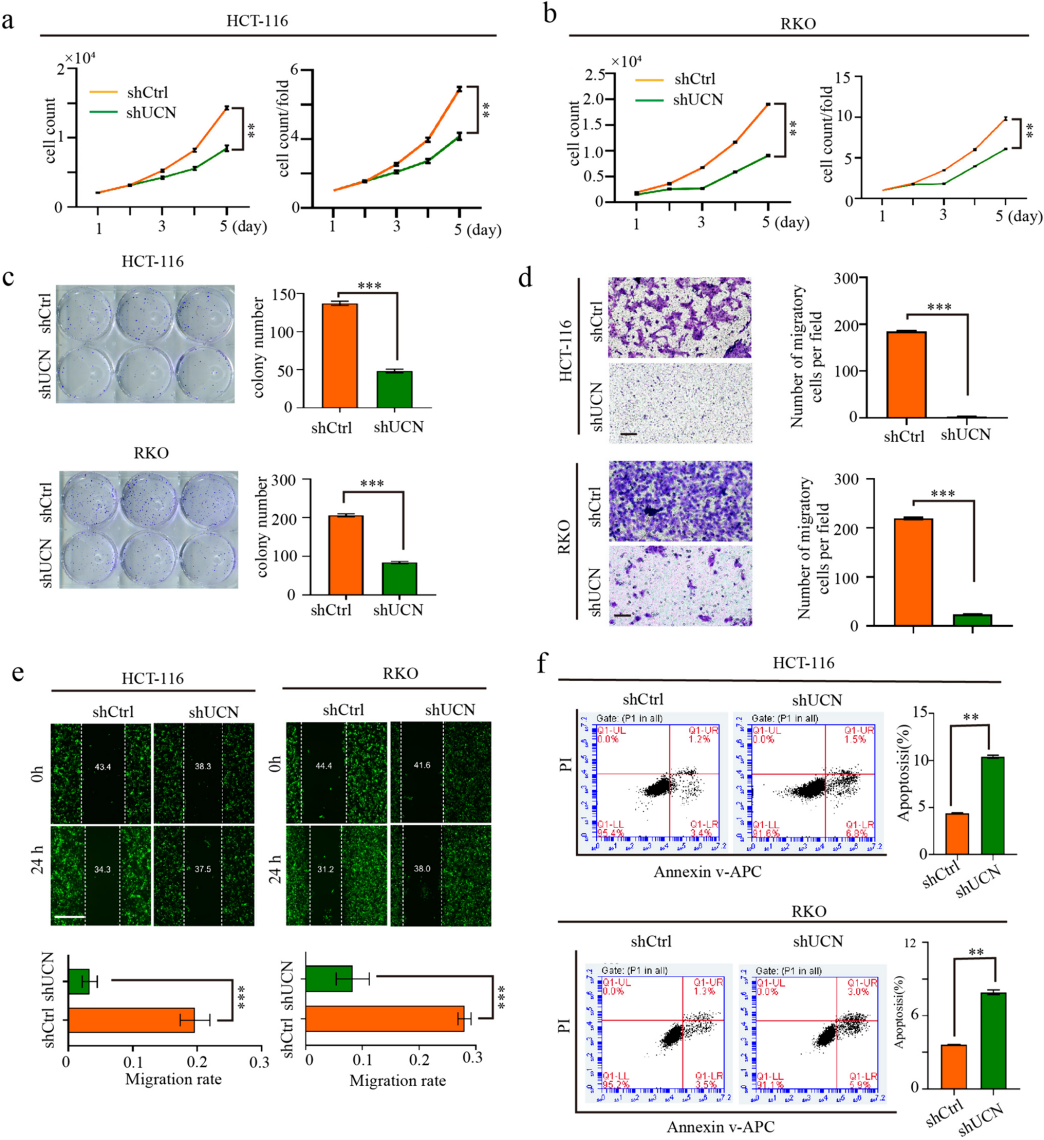
Knockdown of UCN-1 influenced the proliferation, colony formation and apoptosis of HCT-116 cells and RKO cells. **a** and **b** The proliferation of HCT-116 cells (**a**) and RKO cells (**b**) after transfected with shUCN or shCtrl. Data are presented as mean ± SEM. **P < 0.01. *t* test. *n* = 3. **c** The colony formation of HCT-116 cells and RKO cells after infected with shUCN or shCtrl. *t* test. Data are presented as mean ± SEM. ***P < 0.001. *n* = 3. **d** and **e** The migration of HCT-116 cells and RKO cells after transfected with shUCN or shCtrl. Scare bar, 100 μm in (**d**), 1 mm in (**e**). *t* test. Data are presented as mean ± SEM. ***P < 0.001. *n* = 3. **f** The apoptosis of HCT-116 cells and RKO cells after infected with shUCN or shCtrl. *t* test. Data are presented as mean ± SEM. **P < 0.01. *n* = 3

The results of the Celigo cell counting assay demonstrated that the proliferative abilities of HCT-116 and RKO cells were weakened after UCN-1 knockdown (Fig. 3a and b and Fig. S2). The results of the colony-forming assay further confirmed that the suppression of UCN-1 expression attenuated CRC cell proliferation (Fig. 3c). In addition, knockdown of UCN-1 obviously attenuated the migration of HCT-116 and RKO cells based on the results of the Transwell and wound healing assays (Fig. 3d and e). Furthermore, we found that UCN-1 knockdown not only markedly suppressed migration and proliferation but also promoted cell apoptosis. Compared with the controls, the apoptotic rates of HCT-116 cells with UCN-1 knockdown increased from 4.40% ± 0.04% to 10.40% ± 0.20% (Fig. 3f), whereas the apoptotic rates of RKO cells under the same condition increased from 3.64% ± 0.02% to 7.92% ± 0.31% (Fig. 3f). These results indicate that UCN-1 may promote proliferation and migration but suppresses apoptosis in CRC cells.

### Knockdown of UCN-1 inhibited CRC growth in vivo

**Fig. 4:**
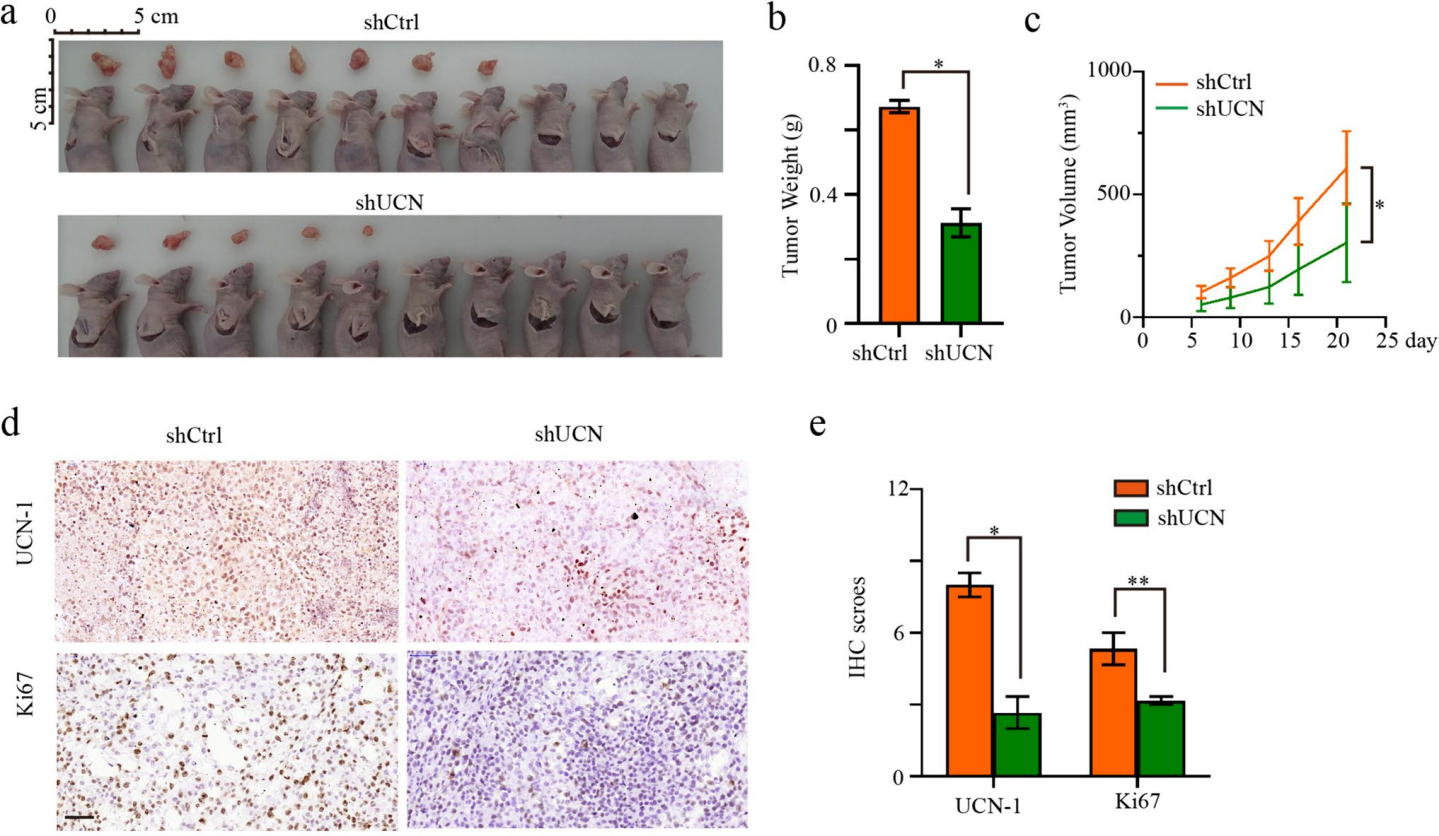
Knockdown of UCN-1 influenced the development of colorectal cancer in vivo. **a** The photos of tumors removed from animal models. **b** The weight of CRC tumors between groups. *t* test. Data are presented as mean ± SEM. *P < 0.05. *n* = 7 in shCtrl group and *n* = 5 in shUCN group. **c** The volume of CRC tumors. *t* test. *n* = 10. *n* = 7 in shCtrl group and n = 5 in shUCN group. **d** Immunohistochemical images of tumor cell proliferation (Ki-67) and UCN-1 in shCtrl and shUCN group. Scale bar, 50 μm. **e** IHC score of Ki-67 and UCN. two-way ANOVA with Tukey’s post hoc multiple comparisons test. Data are presented as mean ± SEM. *P < 0.05, **P < 0.01. *n* = 3

To analyze the effect of UCN-1 knockdown on CRC development in vivo, we established the nude mouse xenograft model using RKO cells. Mice injected with RKO cells with and without UCN-1 knockdown served as the experimental (shUCN) and control group (shCtrl), respectively. In vivo imaging was performed 5–7 days after injection, and the mice were euthanized on day 28. The tumor volume and weight were measured. Tumors of variable sizes were present in both the shCtrl and shUCN groups (Fig. 4a). We found that fewer tumors had formed in the shUCN than in the shCtrl group (Fig. 4a). The tumors in the shCtrl group were ~ 2.15-fold heavier and twofold larger than those in the shUCN group (Figs. 4b and c). Histologically, the protein expression of UCN-1 in the tumors formed in the shUCN group decreased significantly compared with that in the shCtrl group, suggesting that our knockdown strategy was successful (Fig. 4d). The morphology of the tumor cells and the internal structure of the tumors formed in shUCN groups did not differ from controls, although the size and weight of tumors were reduced (Fig. S3). To observe tumor growth, the staining patterns for Ki-67, a proliferation marker, were observed in both groups (Fig. 4d and e). Based on Ki-67 staining, the proliferation rate was significantly higher in the shCtrl than in the shUCN tumors (Fig. 4d and e), indicating that UCN-1 knockdown decreased the proliferation ability of CRC cells in vivo.

### The p53 signaling pathway participated in UCN-1-mediated CRC development

**Fig. 5:**
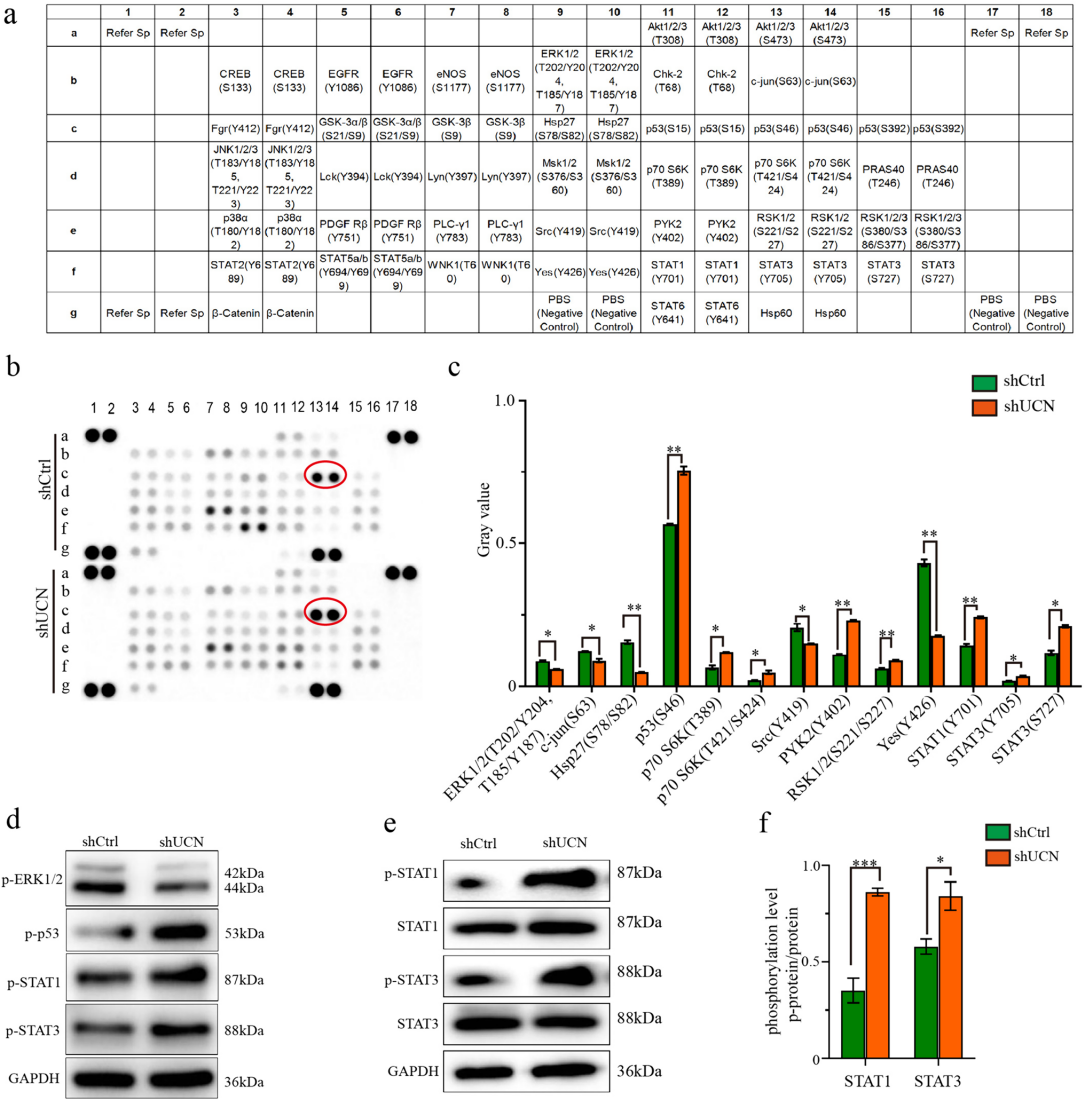
Human phosphor-kinase array was used to identify the downstream pathway of UCN-1 regulating CRC. Distribution of targets and phosphorylation site description. **b** The results of chemiluminescence raw in shCtrl and shUCN group. **c** The statistic chart of (**b**). Data are presented as mean ± SEM. *P < 0.05, **P < 0.01. *n* = 3. P53(S46) was indicated by red circle in (**b**). **d** The results of western blotting in shCtrl and shUCN group. **e** The phosphorylation level of STAT1 (Y701) and STAT3(Y705) in shCtrl and shUCN group. **f** Data are presented as mean ± SEM. *P < 0.05, ***P < 0.001. *n* = 3

To further explore the downstream pathway underlying the regulation of CRC by UCN-1, phosphokinase protein chip technology was used in the RKO cell lines with UCN-1 knockdown. Our results demonstrated that UCN-1 knockdown increased the expression of p53 (S46), p70S6K (T389), p70S6K (T4211/T424), PYK2 (Y402), RSK1/2 (S221/S227), STAT1 (Y701), STAT3 (Y705), and STAT3 (S727). Conversely, the expression of p-kinases, including ERK1/2 (T202/Y204, T185/Y187), c-jun (S63), Hsp27 (S78/S82), Src (Y419) and Yes (Y426) decreased (Fig. 5a–c). Then, the phosphorylation level of p53 (S46), ERK1/2 (T202/Y204, T185/Y187), STAT1 (Y701) and STAT3 (S727) were test by using western blotting, which was consistent with the result of phosphokinase protein chip technology (Fig. 5d). Moreover, p-STAT1 and p-STAT3 with the total STAT1 and STAT3 were quantified by using western blotting, respectively. The phosphorylation level of STAT1 (Y701) and STAT3 (S727) increased greatly after UCN-1 knockdown (Fig. 5e and f).

**Fig. 6:**
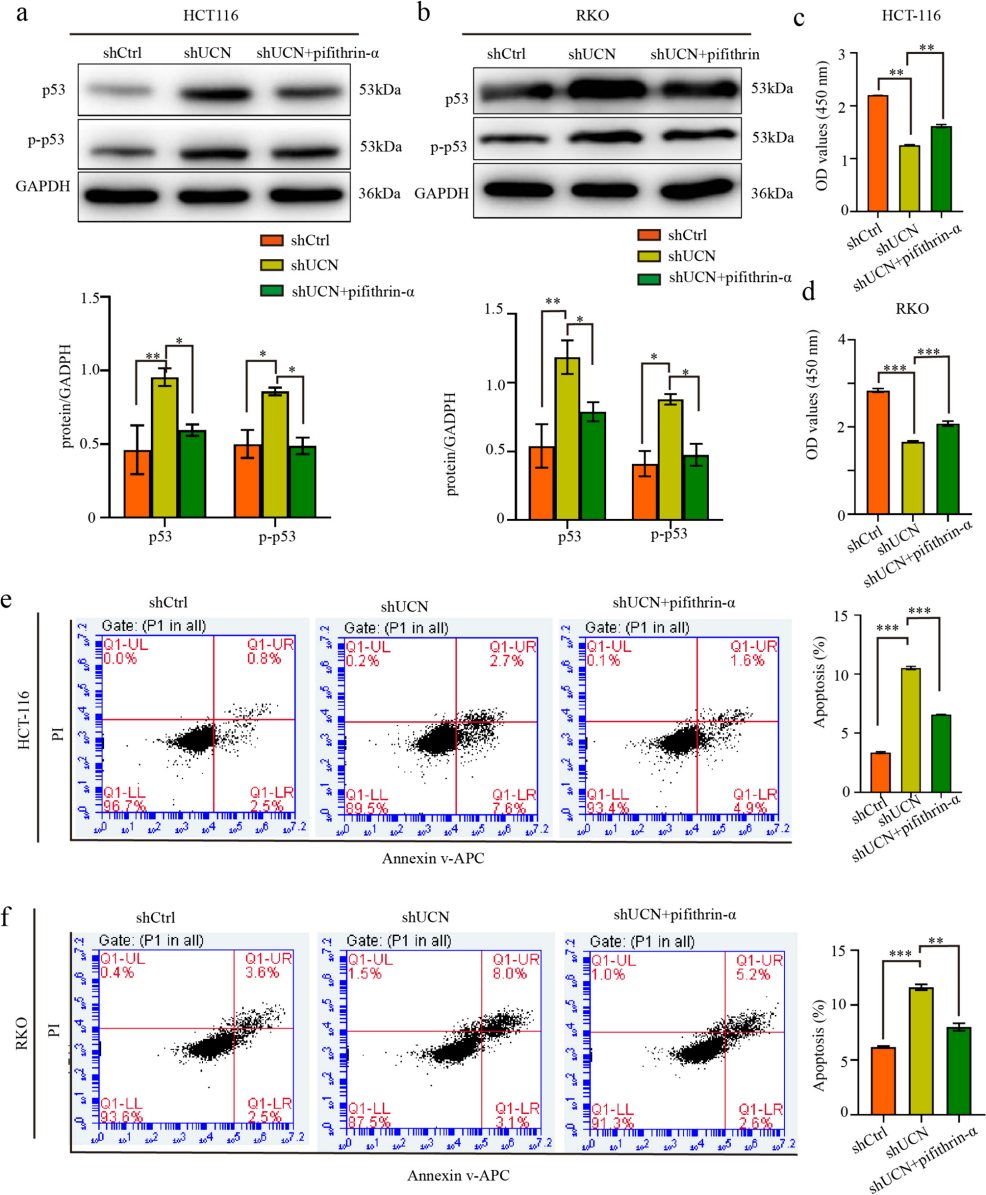
p53 signaling pathway involved in the UCN-1 mediated CRC development. **a** and **b** Protein expression of p53 and p-p53 in HCT-116 cells (**a**) and RKO cells (**b**). two-way ANOVA with Tukey’s post hoc multiple comparisons test. Data are presented as mean ± SEM. *P < 0.05, **P < 0.01. *n* = 3. **c** and **d** The proliferation HCT-116 cells (**c**) and RKO cells (**d**) among groups. One-way analysis of ANOVA with Tukey’s test. Data are presented as mean ± SEM. **P < 0.01, ***P < 0.001. *n* = 3. **e** and **f** The apoptosis of HCT-116 cells (**e**) and RKO cells (**f**) among groups. One-way analysis of ANOVA with Tukey’s test. Data are presented as mean ± SEM. **P < 0.01, ***P < 0.001. *n* = 3

CRC represents the cancer entity with the highest prevalence of p53 mutations (Zhou et al. 2022a, b; Li et al. 2015), indicating that it is associated with the p53 signaling pathway. The phosphorylation level of p53 at Ser46 (p-p53) increased greatly after UCN-1 knockdown (Fig. 5), which was further supported by the western blotting results (Fig. 6a and 6b). Based on these findings, we hypothesized that UCN-1 is negatively correlated with p-p53 and may regulate CRC progression through the inactivation of the p53 signaling pathway. To explore this hypothesis, pifithrin-α (20 µM), a small-molecule inhibitor of the p53 pathway was used. Compared with controls, the protein expression of p53 and p-p53 in the shUCN group increased initially but decreased again after pifithrin-α treatment (Fig. 6a and 6b). Not only protein expression but also the proliferation rates of HCT-116 and RKO cells were affected. The proliferation of HCT-116 and RKO cells decreased by UCN-1 knockdown but the effect was reversed by pifithrin-α treatment (Fig. 6c and d). The apoptosis rate of HCT-116 and RKO cells in the shUCN group increased from 3.37% ± 0.12% and 6.19% ± 0.16% to 10.52% ± 0.25% and 11.62% ± 0.47%, respectively. With pifithrin-α treatment, the apoptosis rate of HCT-116 and RKO cells decreased to 6.57% ± 0.05% and 8.01% ± 0.56%, respectively (Fig. 6e and f). In summary, the effects on proliferation and apoptosis caused by UCN-1 knockdown were reversed after application of an inhibitor of the p53 signaling pathway, indicating that UCN-1 promotes CRC cell proliferation and suppresses apoptosis by inhibiting p53 signaling in vitro.

**Fig. 7:**
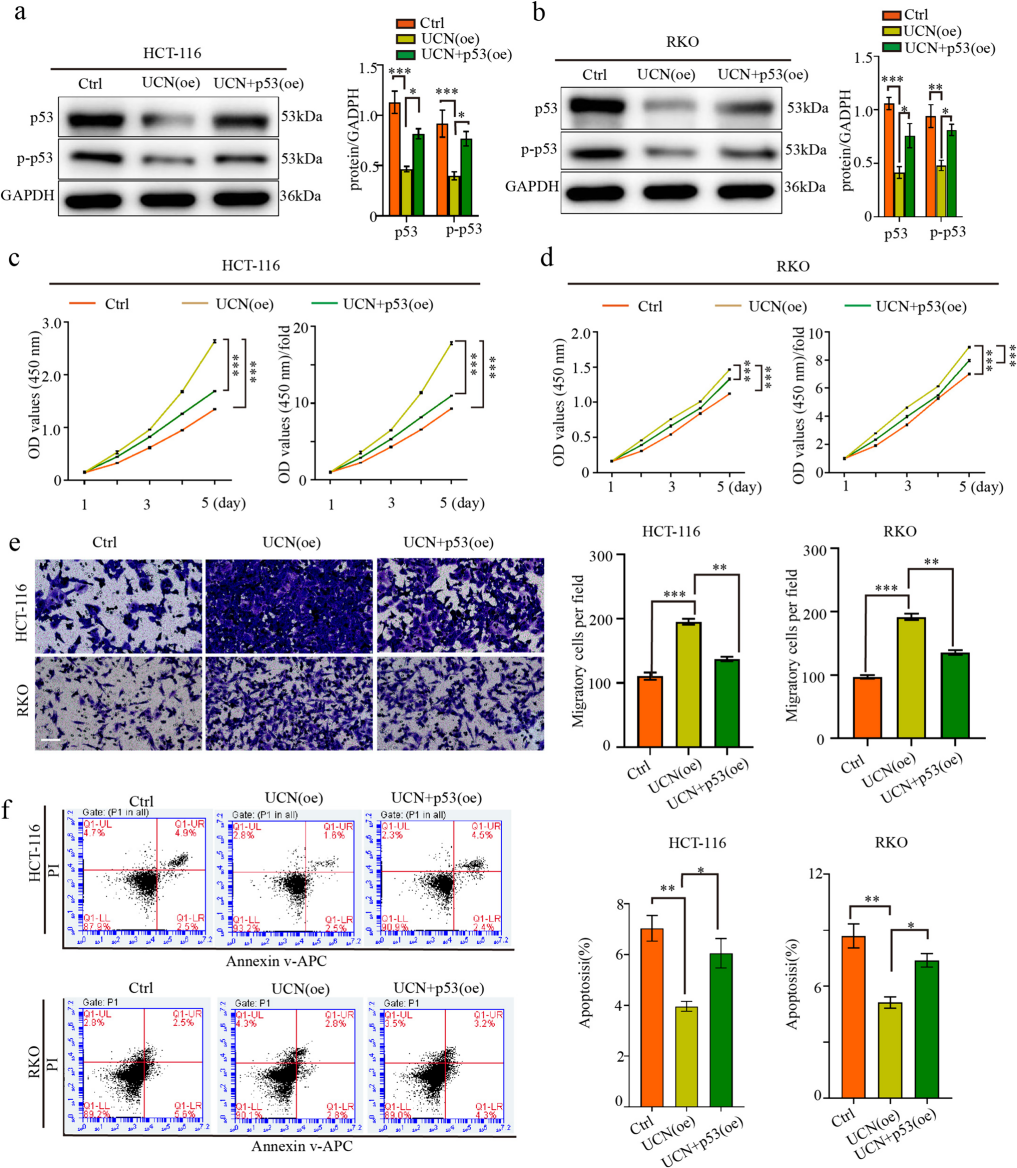
Over-expression of p53 pathway reserved the effect of UCN-1 over-expression on HTC-116 and RKO cells. **a** and **b** Protein expression of p53 and p-p53 in HCT-116 cells and RKO cells. two-way ANOVA with Tukey’s post hoc multiple comparisons test. Data are presented as mean ± SEM. **P < 0.01, ***P < 0.001. *n* = 3. **c** and **d** The proliferation of HCT-116 cells (**c**) and RKO cells (**d**) among groups. One-way analysis of ANOVA with Tukey’s test. Data are presented as mean ± SEM. *P < 0.05, **P < 0.01. *n* = 3. **e** The migration of HCT-116 cells and RKO cells among groups. Scare bar, 100 μm. One-way analysis of ANOVA with Tukey’s test. Data are presented as mean ± SEM. **P < 0.01, ***P < 0.001. *n* = 3. **f** The apoptosis of HCT-116 cells and RKO cells among groups. One-way analysis of ANOVA with Tukey’s test. Data are presented as mean ± SEM. *P < 0.05. *n* = 3

**Fig. 8:**
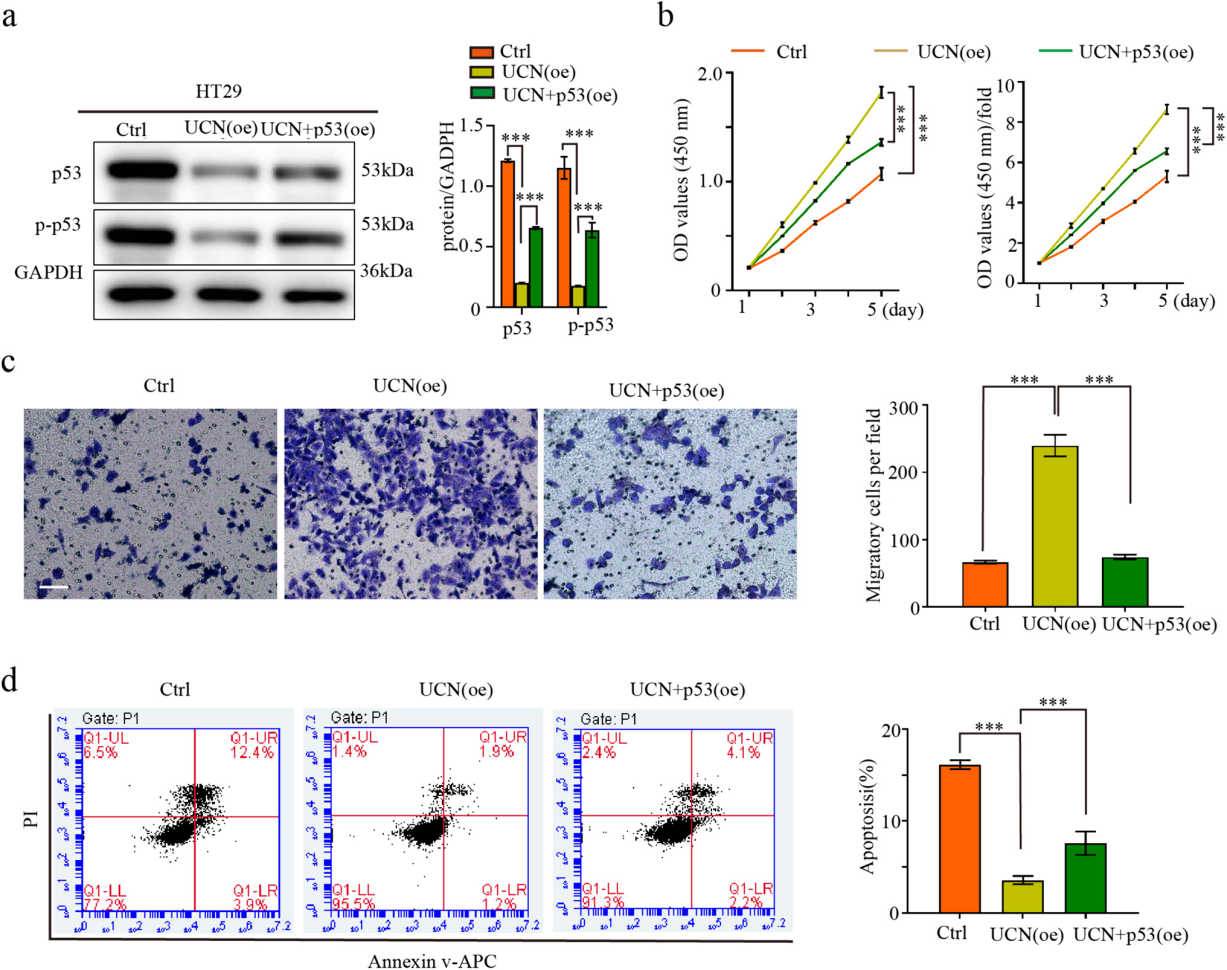
Over-expression of p53 pathway reserved the effect of UCN-1 over-expression on TH29 cells. **a** Protein expression of p53 and p-p53 in HT29 cells. two-way ANOVA with Tukey’s post hoc multiple comparisons test. Data are presented as mean ± SD. **P < 0.01, ***P < 0.001. *n* = 3. **b** The proliferation of HT29 cells among groups. One-way analysis of ANOVA with Tukey’s test. Data are presented as mean ± SEM. *P < 0.05, **P < 0.01. *n* = 3. (**c**) The migration of HT29 cells among groups. Scare bar, 100 μm. One-way analysis of ANOVA with Tukey’s test. Data are presented as mean ± SD. **P < 0.01, ***P < 0.001. *n* = 3. (**f**) The apoptosis of HT29 among groups. One-way analysis of ANOVA with Tukey’s test. Data are presented as mean ± SD. *P < 0.05. *n* = 3

To confirm the above conclusion, we overexpressed UCN-1 using a lentiviral cDNA approach HCT-116 and RKO cells. The protein expression of p53 and p-p53 decreased in the UCN (oe) group but re-induced in the UCN (oe) + p53(oe) group (Fig. 7a and b). The proliferation and migration of the HCT-116 and RKO cells were upregulated by UCN-1 overexpression (Fig. 7c–e and Fig. S4) and reversed in the UCN (oe) + p53(oe) group (Fig. 7c–e). The apoptotic rates of the HCT-116 and RKO cells in the UCN-1 (oe) group decreased from 7.03% ± 0.87% and 8.69% ± 1.11% to 3.96% ± 0.33% and 5.13% ± 0.52%, respectively (Fig. 7f) and re-increased to 6.05% ± 1.01% and 7.38% ± 0.62%, respectively, in the UCN-1 (oe) + p53 (oe) group (Fig. 7f). We performed similar experiments using a lentiviral cDNA approach in HT-29 cells, considering the lowest expression of UCN-1. Compared with controls, the protein expression of p53 and p-p53 decreased in the UCN-1 overexpression group (UCN [oe]) but re-induced by the p53 overexpressed plasmid (UCN [oe] + p53 [oe]) (Fig. 8a). Besides protein expression, the proliferation and migration of HT-29 cells were upregulated after UCN-1 overexpression (Fig. 8b and c). This phenomenon was reversed in the UCN (oe) + p53 (oe) group (Fig. 8b and 8c). The apoptotic rate of the HT29 cells in the UCN-1 (oe) group decreased from 16.14% ± 0.48% to 3.57% ± 0.43% (Fig. 8d) and re-increased to 7.57% ± 1.27% in the UCN-1 (oe) + p53 (oe) group (Fig. 8d). The above results indicate that UCN-1 overexpression promotes CRC cell proliferation and migration and inhibits apoptosis. This function was reserved by overexpression of p53, indicating that the function of UCN-1 in promoting proliferation and migration and decreasing apoptosis in CRC cells are inhibited by overexpression of p53 signaling pathway in vitro.

## Discussion

In this study, we demonstrated that UCN-1 participates in CRC development by inhibiting the p53 signaling pathway. Both the TCGA database and tissue microarray analyses showed overexpression of UCN-1 in patients with CRC. The tissue microarray analysis confirmed the importance of UCN-1 regarding CRC stage, sex, and lymphatic metastasis. Based on these findings, we further investigated the biological functions of UCN-1 in CRC progression. We found that UCN-1 acts as a crucial regulator in CRC development and may significantly enhance the proliferation and migration abilities of CRC cells, as well as suppress cell apoptosis. In the xenograft tumor model experiment, UCN-1 facilitated tumor outgrowth. Next, we used the human phosphorylation array to explore the detailed mechanism and found that p53 acts as a downstream signaling pathway. The suppression in proliferation and migration and increase of cell apoptosis caused by UCN-1 knockdown were reversed after treatment with pifithrin-α. In contrast to UCN-1 knockdown, overexpression of UCN-1 promoted proliferation and migration and decreased cell apoptosis. This effect was reserved by p53 overexpression. Therefore, UCN-1 promotes CRC cell proliferation and migration and restrains apoptosis by inhibiting the p53 signaling pathway.

Numerous studies have revealed that UCN-1 is involved in the development of several cancer types (Balogh et al. 2022; Owens et al. 2017; Kamada et al. 2012) but its contribution to CRC development had not been elucidated and the relationship between CRC and UCN-1 remained unclear. Our study found that UCN-1 was overexpressed in CRC and positively correlated with malignancy based on the immunohistochemical analysis and TCGA database. The biological actions of UCN-1 are exerted through interactions with two distinct CRH receptor subtypes, CRH receptor 1 (CRHR1) and CRH receptor 2 (CRHR2). Both CRH receptors and UCN-1 are distributed in the colon and play an important role in stimulating colonic motor activity (Maillot et al. 2000, 2003; Martinez et al. 2002; You et al. 2015; Saruta et al. 2004). These findings indicate that UCN-1 is expressed in the colon and is associated with CRC. On the other hand, the tissue microarray analysis showed that UCN-1 expression was associated with sex. High UCN-1 expression was more frequently observed in male than in female patients. Interestingly, the incidence of CRC is higher in men than in women (Lewandowska et al. 2022). This result indicates that UCN-1 may be an important factor involved in the correlation between sex and CRC. This is an interesting and meaningful discovery because UCN-1 is not a sex hormone. Why high UCN-1 expression is more likely in male patients? One possible explanation is that UCN-1 and sex hormones are inter-regulated. The distribution of UCN-1 in the nucleus of the solitary tract decreases depending on sex and estrogen levels (Ciriello 2015). Deletion of CRHR1 selectively impairs maternal, but not intermale, aggression (Gammie et al. 2008). These studies indicate that the presence of estrogen can lead to decreased distribution of UCN-1 in some tissues and that UCN-1 affects estrogen secretion. Furthermore, high CRC risk and mortality are more likely in men (Lu et al. 2020). The relationship between UCN-1 and estrogen may explain sex differences in CRC incidence.

Although the underlying mechanism is unknown, these observations may be an interesting research direction regarding the mechanism by which UCN-1 regulates CRC. Metastasis in the lymph nodes is common. Our tissue microarray analysis showed that UCN-1 expression was associated with lymphatic metastasis, indicating that UCN-1 may be related to the ability of CRC cells to migrate. UCN-1 affects the migration of hepatic cancer cells (Zeng et al. 2016) and endometrial cancer cells in vitro (Brenner et al. 2014). Its effect on tumor migration may be related to tumor type. For example, UCN-1 increases the migration of hepatic cancer cells (Zhu et al. 2014) but suppresses the migration ability of endometrial cancer cells (Owens et al. 2017).

CRC cells were used to explore the role of UCN-1 in migration. Our results showed that knockdown of UCN-1 suppressed, while overexpression of UCN-1 enhanced migration ability. To ensure experimental rigor, we used two CRC cell lines, RKO and HCT-116 cells. Knockdown of UCN-1 reduced the migration rate of these two cell lines by 71.4% and 84.2%, respectively. We also investigated the effects of UCN-1 on the proliferation and apoptosis of CRC cells. Knockdown of UCN-1 decreased the proliferation rate and increased the apoptotic rate of CRC cells. This result was verified in animal experiments. UCN-1 knockdown reduced the number and size of tumors formed in nude mice. These results indicate that UCN-1 may act as a target for CRC therapy.

Our results showed that UCN-1 is involved in CRC, but how UCN-1 modulates CRC remained unclear. To address this problem, we used a phosphokinase protein microarray. UCN-1 knockdown increased the expression of p53 and p-p53. p53 is an important tumor suppressor with a role in preventing cancer development. Notably, the p53 signaling pathway has been associated with CRC (Liu et al. 2022, 2021). An interesting phenomenon was observed in our experiment. Phosphorylation of p53 at Ser 46 significantly increased after UCN-1 knockdown, but the phosphorylation levels at Ser 15 and Ser 392 did not change significantly. It is well established that the enhanced stability and transcriptional activity of p53 depend on phosphorylation modification. p53 has multiple phosphorylation sites, including Ser15 and Ser46 in the N-terminal domain and Ser392 in the C-terminal domain. The phosphorylated p53 at different sites plays different physiological roles, with phosphorylation at Ser 46 mainly involved in apoptosis (Liebl and Hofmann 2019). The upregulated phosphorylation of p53 at Ser 46 after UCN-1 knockdown indicated that UCN-1 might suppress apoptosis by promoting the phosphorylation of p53 at Ser46. As expected, after treatment with pifithrin-α, the increase in p53 phosphorylation at Ser 46 and cell apoptosis caused by the UCN-1 knockdown were revoked. Overexpression of p53 also significantly attenuated the suppression of p-p53 at Ser 46 and the apoptosis caused by UCN-1 overexpression. Therefore, UCN-1 is involved in CRC cell apoptosis by promoting the levels of phosphorylation of p53 at Ser46. On other hand, UCN-1 knockdown suppressed the proliferation and migration of CRC cells and treatment with pifithrin-α reserved the suppression of migration and proliferation caused by UCN-1 knockdown. Similarly, the promoting effects of UCN-1 overexpression on migration and proliferation were reserved by overexpression of p53. However, the phosphorylation of p53 at Ser46 has not been reported to mediate cell proliferation and migration. These effects on cell proliferation and migration are likely mediated through phosphorylation at other sites. As p53 has more than 30 phosphorylation sites, UCN-1 may regulate proliferation and migration via other sites. In this study we focused on three phosphorylation sites; thus, more experiments are needed to identify the phosphorylation sites involved in proliferation and migration. In addition to the activation of p53 caused by phosphorylation that suppresses the initiation and progression of CRC (Wang et al. 2019), inactivation of p53 by altering p53 regulators or mutations in p53 also occur frequently in CRC (Huang et al. 2018; Zhou et al. 2019). Ablation of mutant p53 in CRC inhibits Stat3-mediated tumor growth and invasion (Schulz-Heddergott et al. 2018). Loss of p53 function plays a crucial role in treatment resistance in various neoplastic diseases, including CRC. Although our study did not focus on the effect of p53 mutants on CRC, mutant p53s play an important role in CRC.

We speculated that UCN-1 may inhibit CRC by regulating the immune response via the p53 signaling pathway. Several studies have clearly shown that the CRH signaling pathway participates in chronic intestinal disorders, including inflammatory bowel disease (IBD) (Buckinx et al. 2011; Chatoo et al. 2018). Immune imbalance (Chang et al. 2011), such as that observed in IBD (Keller et al. 2019; Mattar et al. 2011), is considered a predisposition factor for developing CRC. UCN-1 modulates the gastrointestinal tract by regulating the intestinal immune environment (Balkwill and Mantovani 2001). On the other hand, p53 has been reported to regulate the inflammatory tumor microenvironment (Uehara and Tanaka 2018) and immune factors (Cooks et al. 2018; Li et al. 2020). Based on these studies, UCN-1 may inhibit CRC by regulating the immune response via the p53 signaling pathway. This aspect was not addressed in this study and will be a direction for our follow-up studies.

In summary, our study demonstrated the function of UCN-1 in CRC development and explored whether UCN-1 modulated CRC via the p53 signaling pathway. We found that high expression of UCN-1 was likely associated with patients with CRC with a worse pathological prognosis. Knockdown of UCN-1 decreased, but overexpression of UCN-1 increased, migration and proliferation in vitro. Besides, knockdown of UCN-1 induced apoptosis in vitro. In vivo, knockdown tumors were smaller and less proliferative than controls. Furthermore, the effect of UCN-1 knockdown on CRC development was reversed by inhibition of the p53 signaling pathway. On the other hand, UCN-1 overexpression increased proliferation and migration and decreased apoptosis. This effect was reversed by p53 overexpression. Based on the results of our study, UCN-1 participates in the regulation of CRC progression via inhibition of the p53 signaling pathway. This finding has not been reported previously and may provide a new target for CRC treatment.

## Supplementary Information

Below is the link to the electronic supplementary material.

Supplementary file1 (DOCX 411 KB)

## Data Availability

The datasets generated during the current study are not publicly available. Because this is a basic study and data sharing is not necessary. But they are available from the corresponding author on reasonable request.
