# Supplementary material for: Urocortin-1 promotes colorectal cancer cell migration and proliferation and inhibits apoptosis via inhibition of the p53 signaling pathway
Source: J Cancer Res Clin Oncol. 2024 Mar 28;150(3):163. doi: 10.1007/s00432-024-05693-7 (PMC10978644; doi:10.1007/s00432-024-05693-7)
Supplement: Supplementary file 1 — Supplementary file1 (DOCX 411 KB) [file 432_2024_5693_MOESM1_ESM.docx]

**Urocortin-1 Stimulates Colorectal Cancer Cells Migration, Proliferation and Invasion via p53 Signaling Pathway**

**Xiaolan Guo, Ya Li, Xiangyu Chen, Binghua Sun and Xiaolan Guo^1^***

^1^ Department of Gastroenterology, the First Affiliated Hospital of Zhengzhou University, Zhengzhou, Henan, China.

***Correspondence:**gxlzdyfy@163.com

**Keywords: Colorectal cancer, Urocortin-1, p53, HT29, HCT-116, RKO**

**Supplementary Figure 1 | The knockout efficiency of lentivirus.**

The knockdown efficiency of three different shRNA targeting UCN (shUCN_1, shUCN_2 and shUCN_3). Data are presented as mean ± SEM. *P<0.05.

**Supplementary Figure 2 | The effect of knockdown UCN-1 on proliferation of HCT-116 cells and RKO cells.**

(a and b) The proliferation of HCT-116 cells (a) and RKO cells (b) after transfected with shUCN or shCtrl.

**Supplementary Figure 3 | The Tumor morphology of control and UCN-1 knockout groups.** There were no significant differences in HE. Scale bar, 50 μm.

**Supplementary Figure 4 | The effect of overexpression of UCN-1 and p53 on proliferation of HCT-116 cells and RKO cells.**

(a and b) The proliferation of HCT-116 cells (a) and RKO cells (b) after overexpressing UCN-1 and p53.


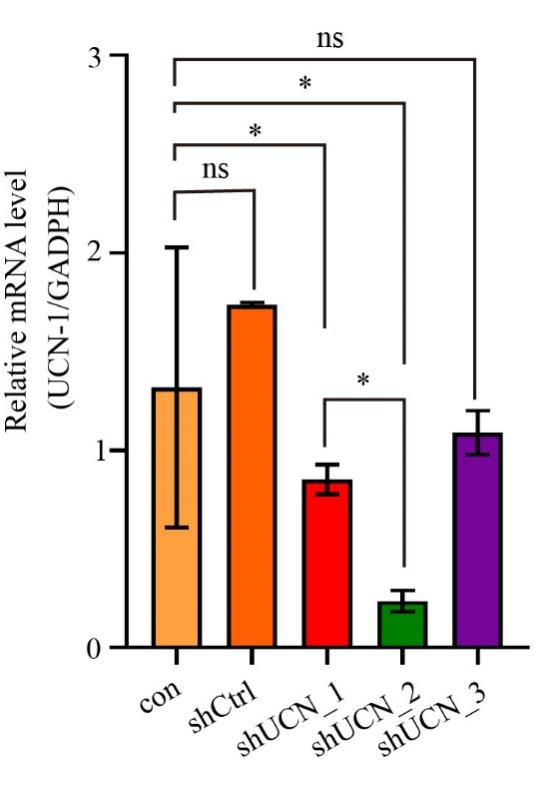


Supplementary Figure 1 | The knockout efficiency of lentivirus.


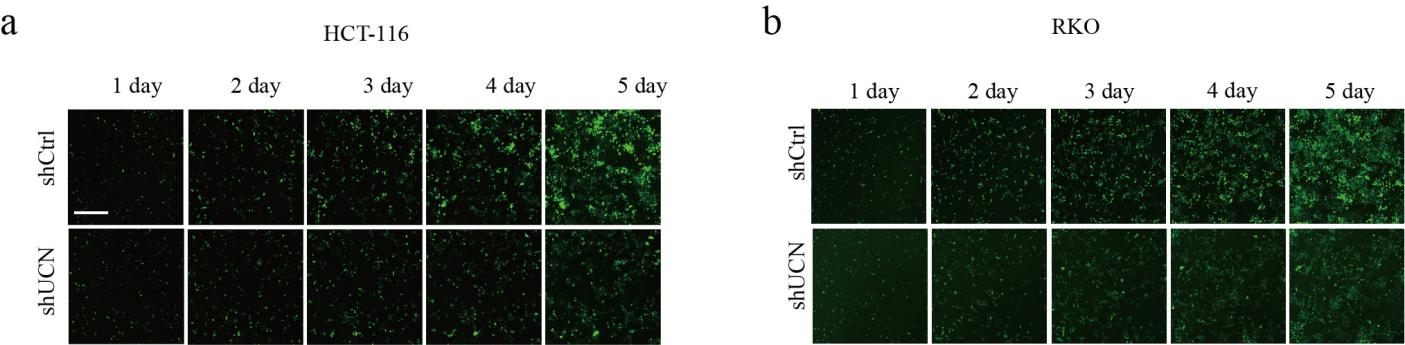


Supplementary Figure 2 | The effect of knockdown UCN-1 on proliferation of HCT-116 cells (a) and RKO cells (b).Scare bar, 500 μm.


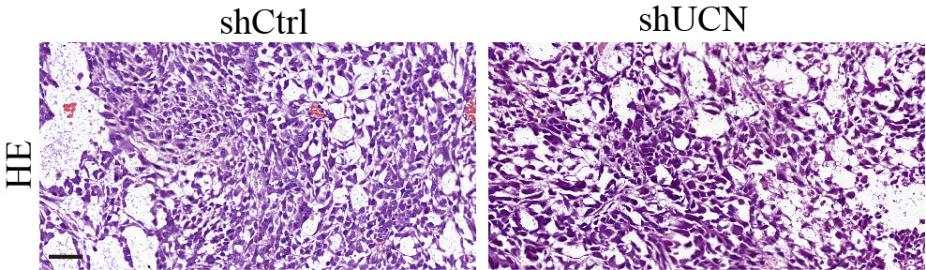


Supplementary Figure 3 | The Tumor morphology of control and UCN-1 knockout groups. Scare bar, 100 μm.

**
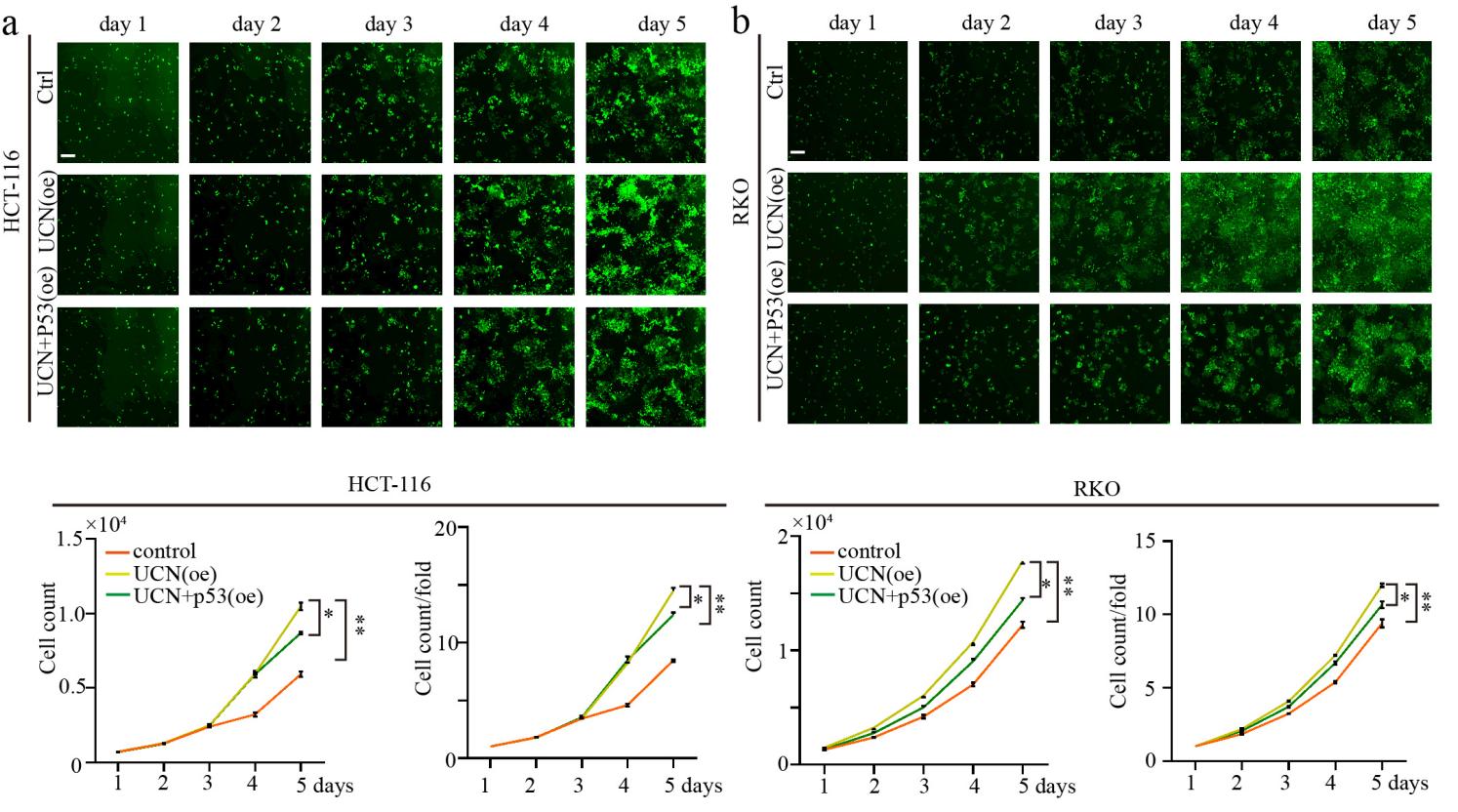
**

Supplementary Figure 4 | The effect of overexpression of UCN-1 and p53 on proliferation of HCT-116 cells and RKO cells. Scare bar, 200 μm.
